# Managing the Risk of Lung Toxicity with Trastuzumab Deruxtecan (T-DXd): A Canadian Perspective

**DOI:** 10.3390/curroncol30090582

**Published:** 2023-08-30

**Authors:** Jan-Willem Henning, Christine Brezden-Masley, Karen Gelmon, Stephen Chia, Shane Shapera, Micheal McInnis, Daniel Rayson, Jamil Asselah

**Affiliations:** 1 Tom Baker Cancer Centre, 1331-29th Street, Calgary, AB T2N 4N2, Canada; 2 Mount Sinai Hospital, 1284-600 University Avenue, Toronto, ON M5G 1X5, Canada; christine.brezden@sinaihealth.ca; 3 BC Cancer Agency, 600 10th Avenue West, Vancouver, BC V5Z 4E6, Canada; kgelmon@bccancer.bc.ca (K.G.); schia@bccancer.bc.ca (S.C.); 4 University Health Network, University of Toronto, Toronto General Hospital, 9N-971, 585 University Avenue, Toronto, ON M5G 2N2, Canada; shane.shapera@uhn.ca; 5 Department of Medical Imaging, University of Toronto, 585 University Ave, Toronto, ON M5G 2N2, Canada; micheal.mcinnis@uhn.ca; 6 Department of Medical Oncology, Dalhousie University, QEII-Bethune Building, 1276 South Park Street, Halifax, NS B3H 2Y9, Canada; daniel.rayson@nshealth.ca; 7 Cedars Cancer Centre, McGill University Health Centre, 1001 Decarie Boulevard, Montreal, QC H4A 3J1, Canada; jamil.asselah@mcgill.ca

**Keywords:** trastuzumab deruxtecan, metastatic breast cancer, interstitial lung disease, pneumonitis

## Abstract

Ongoing advances in precision cancer therapy have increased the number of molecularly targeted and immuno-oncology agents for a variety of cancers, many of which have been associated with a risk of pulmonary complications, among the most concerning being drug-induced interstitial lung disease/pneumonitis (DI-ILD). As the number of patients undergoing treatment with novel anticancer agents continues to grow, DI-ILD is expected to become an increasingly significant clinical challenge. Trastuzumab deruxtecan (T-DXd) is an antibody–drug conjugate targeting human epidermal growth factor receptor 2 that is gaining widespread use in the metastatic breast cancer setting and is undergoing exploration for other oncologic indications. ILD/pneumonitis is an adverse event of special interest associated with T-DXd, which has potentially fatal consequences if left untreated and allowed to progress. When identified in the asymptomatic stage (grade 1), T-DXd-related ILD can be monitored and treated effectively with the possibility of treatment continuation. Delayed diagnosis and/or treatment, however, results in progression to grade 2 or higher toxicity and necessitates immediate and permanent discontinuation of this active agent. Strategies are, therefore, needed to optimize careful monitoring during treatment to ensure patient safety and optimize outcomes. Several guidance documents have been developed regarding strategies for the early identification and management of T-DXd-related ILD, although none have been within the context of the Canadian health care environment. A Canadian multidisciplinary steering committee was, therefore, convened to evaluate existing recommendations and adapt them for application in Canada. A multidisciplinary approach involving collaboration among medical oncologists, radiologists, respirologists, and allied health care professionals is needed to ensure the proactive identification and management of T-DXd-related ILD and DI-ILD associated with other agents with a similar toxicity profile.

## 1. Introduction

**Figure 1:**
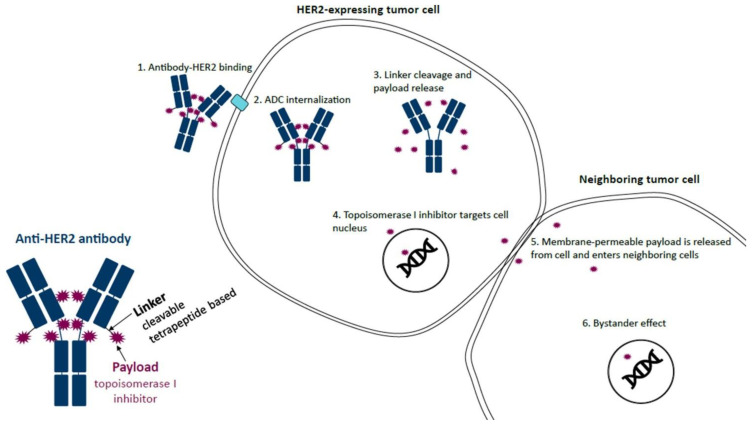
Mechanism of action of trastuzumab deruxtecan (T-DXd). T-DXd is composed of an anti-HER2 antibody, a linker, and a topoisomerase I inhibitor payload. The anti-HER2 antibody binds to HER2 on tumor cells, which leads to ADC internalization. The linker, which is selectively cleaved by cathepsins that are upregulated in cancer cells, is then cleaved and releases the topoisomerase I inhibitor payload. The topoisomerase I inhibitor enters the nucleus of the cell, resulting in cell death. Upon cell death, the membrane-permeable topoisomerase I inhibitor is released from the cell and can then enter neighboring cells regardless of whether they express HER2. ADC, antibody–drug conjugate; HER2, human epidermal growth factor receptor 2; T-DXd, trastuzumab deruxtecan. Reproduced with permission from Swain et al. [5]. Reprinted from *Cancer Treatment Reviews*, Vol. 106, Swain S.M., et al., Multidisciplinary clinical guidance on trastuzumab deruxtecan (T-DXd)-related interstitial lung disease/pneumonitis-Focus on proactive monitoring, diagnosis, and management, 102378, Copyright (2022), with permission from Elsevier.

A number of systemic agents used for the treatment of cancer have been associated with pulmonary toxicities, one of the most concerning of which is drug-induced interstitial lung disease (ILD)/pneumonitis (DI-ILD). Many novel therapies, including molecularly targeted agents, immune checkpoint inhibitors, and antibody–drug conjugates (ADCs) have been associated with a risk of DI-ILD. As the number of anticancer agents continues to grow, DI-ILD is expected to become an increasingly significant clinical challenge across all types of cancers. The recent approval of trastuzumab deruxtecan (T-DXd), a novel ADC targeting human epidermal growth factor receptor 2 (HER2) (Figure 1), for various lines of therapy and indications in breast cancer has highlighted this issue and is the focus of this review.

ILD has been identified as an adverse event of special interest with T-DXd and occurs in approximately 10% to 14% of breast cancer patients treated with the drug [1,2,3,4]. If not effectively identified and treated, T-DXd-related ILD can be fatal. While asymptomatic (grade 1) DI-ILD caused by most drugs does not require specific therapy, T-DXd-related ILD is unique because of the high risk of evolution to serious illness (grades 3–4 ILD) and because early identification of grade 1 ILD may allow for ongoing treatment, whereas progression to grade 2 or higher ILD necessitates permanent discontinuation of T-DXd.

Several guidance documents have been developed in the United States and Europe outlining strategies for the early identification and management of T-DXd-related ILD [5,6]. This multidisciplinary steering panel was convened to evaluate existing recommendations in the context of the Canadian health care system with the goal of developing a pragmatic strategy to optimize the therapeutic benefit of T-DXd for Canadian patients.

## 2. Indications for T-DXd in Canada

T-DXd is approved in Canada for the treatment of adult patients with unresectable or metastatic HER2+ breast cancer as a third-line therapy after disease progression following taxanes, trastuzumab + pertuzumab, and trastuzumab-emtansine, as well as for early disease recurrence in the neoadjuvant or adjuvant setting, and for second-line therapy in patients previously treated with taxanes and trastuzumab + pertuzumab [1,2,7]. In 2023, T-DXd was also approved for the treatment of adult patients with unresectable or metastatic HER2-low breast cancer who have received at least two prior lines of endocrine therapy in addition to one line of chemotherapy in the metastatic setting or following disease recurrence during or within 6 months of completion of adjuvant chemotherapy. HER2-low is defined as a score of 1+ on immunohistochemistry (IHC) or an IHC score of 2+ with no HER2 amplification on in situ hybridization [4].

Approvals for these indications were based on significant improvements in all relevant efficacy endpoints compared to standard of care therapy observed in the DESTINY-Breast01 [1,8], DESTINY-Breast02 [2], DESTINY-Breast03 [3], and DESTINY-Breast04 [4] trials (Table 1).

In addition to the indications for metastatic breast cancer, T-DXd is approved in the United States for locally advanced/metastatic HER2-positive gastric cancer, based on the positive results of the DESTINY-Gastric01 and DESTINY-Gastric02 trials [9,10]; for metastatic HER2-positive nonsmall cell lung cancer (NSCLC), based on the DESTINY-Lung01 and DESTINY-Lung02 trials [11,12,13]; and is under investigation for various additional tumor types, including colorectal and other cancers [14,15].

## 3. Known Risk Factors for DI-ILD

The term “ILD/pneumonitis” is broadly used to describe a diverse group of inflammatory lung disorders affecting alveolar structures, pulmonary interstitium, and small airways and is characterized by the presence of inflammation or scarring of lung parenchyma [16]. Identifiable causes include exposure to organic materials, drugs, or toxins that trigger hypersensitivity pneumonitis; exposure to inorganic dusts and other compounds causing pneumoconiosis; autoimmune conditions such as rheumatoid arthritis and scleroderma; uncommon or rare genetic abnormalities (mutations in telomerase enzymes, mucin genes, surfactant proteins, etc.); and exposure to certain drugs [16].

A number of drug classes have been implicated in DI-ILD, including disease-modifying antirheumatic drugs (DMARDs), antiarrhythmics, antimicrobials, and antineoplastic agents [17,18].

Key risk factors that predict for an increased risk of developing DI-ILD include a history of pre-existing lung disease and reduced lung function [18,19,20,21]; poor performance status [22]; smoking [18]; age older than 60 years [18,19,21]; Japanese or African American ethnicity [21,23]; and male sex [18,19]. Specifically related to oncology, prior treatment with multiple chemotherapy regimens or thoracic radiotherapy; history of radiation recall pneumonitis; presence of lung cancer, lung metastases, or other drug-induced pneumonitides; ongoing therapy with multiple molecularly targeted agents; and treatment with a combination of molecularly targeted and cytotoxic agents have all been identified as risk factors predisposing to DI-ILD [18,21,22]. Factors that increase the risk of poor outcomes and/or mortality from DI-ILD include acute symptomatic disease with rapid symptom onset, hypoxemia, need for mechanical ventilation (associated with a mortality rate > 60%), pre-existing ILD, male sex, age over 65 years, and a diagnosis of nonsmall cell lung cancer [24,25,26,27,28,29]. However, it is important to note that many people who develop DI-ILD have no identifiable pre-existing risk factors, which highlights the need for vigilance.

The identification and monitoring of patients at risk of DI-ILD are crucial for timely intervention; however, there are currently no effective strategies for identifying and monitoring DI-ILD in clinical practice beyond CT imaging and monitoring of oxygen saturation. Prospective clinical trials are on the horizon to determine if there are any helpful screening tools. The authors of this paper encourage Canadian clinicians to collect real-world data on the incidence of T-DXd-related ILD and other potential adverse events.

## 4. DI-ILD with Specific Anticancer Treatments

DI-ILD has been recognized as an important toxicity associated with a number of chemotherapeutic and targeted antineoplastic therapies (Table 2). Bleomycin is the historical example, with a reported incidence of up to 45% and up to a 3% mortality rate [5,30,31]. Contemporary examples include agents targeting mammalian target of rapamycin (mTOR) [32,33], tyrosine kinase/anti-epidermal growth factor receptor (EGFR) inhibitors [34,35,36,37,38,39,40,41], anti-HER2 agents [42,43,44,45,46], *BRAF* inhibitors [47], cyclin-dependent kinase 4/6 inhibitors [48,49,50], and poly (ADP-ribose) polymerase (PARP) inhibitors [51], as well as immune checkpoint inhibitors [52,53,54,55,56,57] and ADCs [5], with case-fatality rates ranging from 0% to 51.3% depending on the drug [18]. DI-ILD has been reported, to a lesser extent, with other widely used conventional chemotherapeutic agents, such as taxanes and gemcitabine, with an incidence of DI-ILD of up to 5% [58,59], and rare but serious events can arise with oxaliplatin [60].

The pathogenesis of DI-ILD is poorly understood, but several mechanisms—both cytotoxic and immune related—may be involved, either alone or in combination, depending on the drug. Direct damage to pneumocytes or alveolar endothelial cells, cell-mediated lung injury, oxidative stress, and systemic cytokine release may all contribute to DI-ILD [61]. In patients treated with immune checkpoint inhibitors, these mechanisms may be compounded by amplified auto-immune processes triggered by the therapy [57]. Further studies are needed to investigate further the cytotoxic and immune-related mechanisms involved in DI-ILD to provide a better understanding of the underlying processes involved and potentially aid in the development of preventive strategies.

## 5. T-DXd and the Risk of ILD

ILD was first identified as an adverse event of special interest in the DESTINY-Breast01 trial, where 13.6% of patients experienced independently adjudicated ILD and 2.2% died because of this complication [1]. Subsequently, guidelines for the identification and management of ILD were incorporated into the DESTINY clinical trial program with a focus on close monitoring and active management including corticosteroids, along with dose interruption/modification and mandatory discontinuation of T-DXd for grade 2 or higher ILD events.

In a pooled analysis of heavily treated patients across 9 phase 1 and 2 T-DXd clinical trials, the incidence of T-DXd-related ILD was 15.4%, with 11.9% experiencing grade 1 or 2 events and a 2.2% incidence of grade 5 events [62]. Rates of ILD ranged from 10.1% in DESTINY-Gastric02 [10] to 26.4% in DESTINY-Lung01 [11] (Table 3). Most events (87%) occurred during the first 12 months of treatment, with a median time to onset of 5.4 months (range < 0.1 to 46.8 months) overall and of 3.2 months for grade 5 events (range < 0.1 to 20.8 months) [62].

Potential risk factors for DI-ILD in the pooled analysis included baseline oxygen saturation (SpO_2_) < 95%, T-DXd dose > 6.4 mg/kg q3w, >4 years since initial disease diagnosis, renal dysfunction, age < 65 years, and baseline or prior lung comorbidities (asthma, chronic obstructive pulmonary disease (COPD), prior ILD/pulmonary fibrosis, and radiation pneumonitis) [62]. Treatment in Japan was also identified as a risk factor for DI-ILD in the pooled analysis [62]; however, T-DXd was initially studied in Japan without the monitoring protocols implemented in later trials, which may account for the higher incidence in this population.

As with other anticancer agents, the underlying mechanisms of T-DXd-related DI-ILD are unclear, but the proposed pathogenesis includes target-dependent and/or -independent uptake and catabolism of the ADC or a bystander effect of the cytotoxic payload released from cells following ADC catabolism [63]. Lung epithelial cells express HER2 protein, and off-cancer target mechanisms have been suggested on the basis of animal studies which observed localization of T-DXd in alveolar macrophages rather than pulmonary epithelial cells [64]. The release of the chemotherapy payload and subsequent bystander effect resulting in cytotoxic lung injury is currently the leading hypothesis in the understanding of T-DXd-related ILD [5].

## 6. Diagnosis and Monitoring of T-DXd-Related ILD

**Figure 2:**
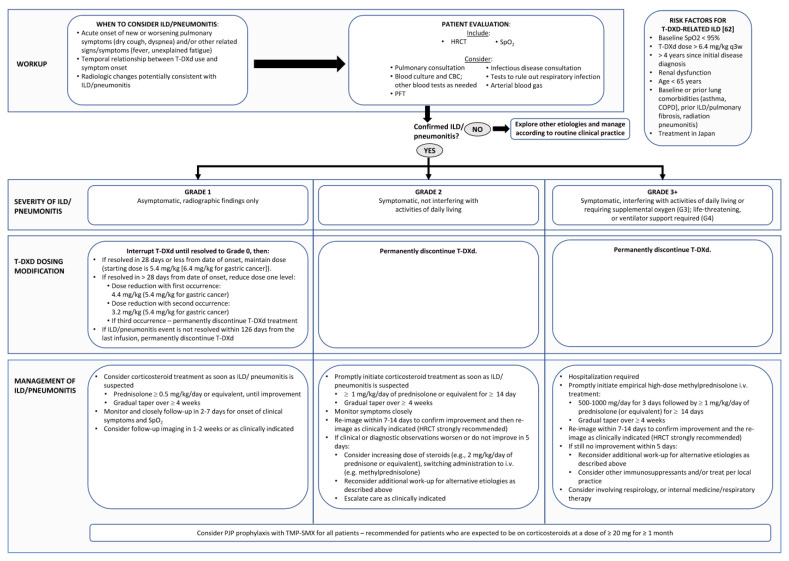
Steering Committee recommendations for the multidisciplinary diagnosis and management of interstitial lung disease/pneumonitis in patients undergoing treatment with trastuzumab deruxtecan (T-DXd). These guidelines have been adapted from guidelines published by Modi et al. [1] and Swain et al. [5] and based on the Canadian product monograph [7]. CBC: Complete blood count; COPD: Chronic obstructive pulmonary disease; HRCT: High resolution computed tomograph; PJP: *P jirovecii* pneumonia; PFT: pulmonary function test; SpO_2_: oxygen saturation.

Current published recommendations for the early identification and management of T-DXd-related ILD in other jurisdictions do not necessarily fully apply to the Canadian health care environment, with variable timely access to pulmonary function tests (PFTs), high-resolution computed tomography (HRCT), and subspecialty respirology expertise. The steering committee therefore sought to tailor existing recommendations and create a practical approach for the Canadian health care landscape (Figure 2).

Diagnosis of DI-ILD requires timely investigation and multidisciplinary collaboration among the oncologist, respirologist, radiologist, and other allied health care providers. In the case of reasonable causality between T-DXd and development of ILD/pneumonitis, prompt diagnosis and therapeutic intervention is key. Other diagnoses (e.g., bacterial, viral, and fungal infections; alveolar hemorrhage; metastases; heart failure; aspiration pneumonia; radiation-induced lung injury; and pulmonary embolism with infarction [65]) should be kept under consideration for atypical cases and nonresponding patients. Opportunistic infections such *Pneumocystis jirovecii* (PJP) should be strongly considered for patients on systemic corticosteroids or other immunosuppressive therapies.

While the risk of T-DXd-related ILD appears to plateau after 12 months [62], it can occur at any time, and long-term monitoring and vigilance are essential.

### 6.1. Key Investigations

#### 6.1.1. Medical History

At baseline, a history and physical examination focusing on known T-DXd-related ILD risk factors should be conducted regardless of the patient’s age. If significant risk factors exist (i.e., baseline SpO_2_ < 95%, T-DXd dose > 6.4 mg/kg q3w, >4 years since initial disease diagnosis, renal dysfunction, age < 65 years, and baseline or prior lung comorbidities), patient discussion should include a risk–benefit ratio of treatment with T-DXd and consideration of alternative agents.

#### 6.1.2. Monitoring for Symptoms

On-treatment clinical visits should focus on careful pulmonary symptom assessment in the context of a functional inquiry and physical examination to detect early signs and symptoms of T-DXd-related ILD. Patients should receive continuing education and regular reminders about the potential adverse events associated with T-DXd, including ILD. At each visit, patients should be asked about any potential symptoms of ILD and be advised of the risk and the need to immediately report symptoms [5]. Symptoms of DI-ILD are nonspecific and include **cough (particularly dry cough), shortness of breath/exertional dyspnea, fever, and unexplained fatigue**.

#### 6.1.3. Pulmonary Function Testing (PFT)

Assessment of SpO_2_ with pulse oximetry should be conducted at baseline and at each pretreatment clinic visit or prior to treatment in the infusion center. An SpO_2_ of <95% or a drop of more than 4% from baseline during treatment is correlated with an increased risk of ILD and ILD severity [62] and should prompt a detailed respiratory assessment.

For patients with new pulmonary symptoms, PFT including spirometry for forced vital capacity (FVC) and diffusing capacity, an exertional pulse oximetry walk test, or six-minute walk test [5] can identify abnormalities associated with pneumonitis and help to quantify the degree of physiological impairment. In particular, a low diffusion capacity (corrected for hemoglobin) can be a sensitive marker of parenchymal lung disease. Various guidelines recommend baseline and follow-up PFT testing for patients on T-DXd [1,5]; however, they may be difficult to interpret in a patient with significant pulmonary metastases, and there are currently **no data to support a reduction in ILD incidence or early detection**. In many centers in Canada, formal PFTs may take several weeks to arrange, and it is essential that decision making is not delayed while awaiting these investigations.

The steering committee recommends considering baseline PFTs only for patients with a history of lung comorbidities (asthma, COPD, prior ILD/pneumonitis, pulmonary fibrosis, pulmonary emphysema, and radiation pneumonitis) with repeat studies to aid in the adjudication of etiology in complex cases. Patients with asthma, COPD, or a history of previous ILD/pneumonitis should be evaluated by their respirologist (or referred for a new respirology consult if they are not already under the care of a respirologist) before starting treatment with T-DXd, if possible.

#### 6.1.4. Chest Imaging

All patients undergoing treatment with T-DXd should undergo a baseline chest CT; the initial oncologic disease assessment CT is sufficient for this purpose. Because certain pre-existing lung conditions are risk factors for DI-ILD, imaging is critical to ensure safe consideration of T-DXd. Documentation of abnormal findings on pretreatment images, with particular attention to evidence of pre-existing ILD, is critical for the assessment of ILD on subsequent CT scans [30].

Patients with metastatic breast cancer on therapy generally undergo conventional CT scans every 9 to 12 weeks, and in many cases this will be sufficient for diagnosis of ILD. If ILD is suspected but not confidently diagnosed on conventional CT, HRCT should be promptly performed to confirm the diagnosis.

In contrast to a routine restaging CT, the HRCT protocol is performed without contrast and includes thinner slices (≤2 mm), as well as expiratory and prone imaging in cases of uncertainty with supine imaging revealing possible dependent atelectasis [66]. Volumetric image acquisition and both coronal and sagittal reconstructions can be helpful in determining the distribution of disease. All modern CT scanners are able to accommodate this protocol (available from the Canadian Society of Thoracic Radiologists: https://car.ca/wp-content/uploads/2020/02/High-Resolution-CT-of-the-Chest-Recommended-Technique-2020.pdf (accessed on 6 February 2023) [66].

In the T-DXd clinical trials, disease and response assessments were conducted every 6 weeks with CT or MRI [67], an interval typically not feasible outside of a clinical trial. Further research is needed to determine the optimal imaging frequency for DI-ILD monitoring. The steering committee recommends CT chest imaging as per routine nontrial protocols, every 9 to 12 weeks, until further evidence becomes available regarding optimal imaging intervals while on T-DXd. HRCT may be used when the conventional CT is suspicious for ILD or when there is clinical suspicion outside of planned disease reassessment CT intervals.

In clinical practice, the CT requisition does not routinely state the specific therapy that a patient with breast cancer is receiving. With T-DXd, it is particularly important that the radiologist be alerted on the imaging requisition that the patient is potentially on a pneumotoxic drug and that the images be examined for both assessment of tumor response as well as for T-DXd-related ILD. The radiologist should be vigilant in identifying inflammatory pulmonary findings, as well as to adjudicate etiology, in the case of nonspecific findings of potential multifactorial origin. The results should be communicated as quickly as possible to ensure that prompt action can be taken.

Although the imaging presentation of DI-ILD is often nonspecific, there are four common patterns of DI-ILD on CT to consider—diffuse alveolar damage (DAD); nonspecific interstitial pneumonia (NSIP); hypersensitivity pneumonitis (HP); and organizing pneumonia (OP) [63]. With T-DXd, OP and HP patterns have been observed and, in some severe cases, DAD [5,68,69].

**Figure 3:**
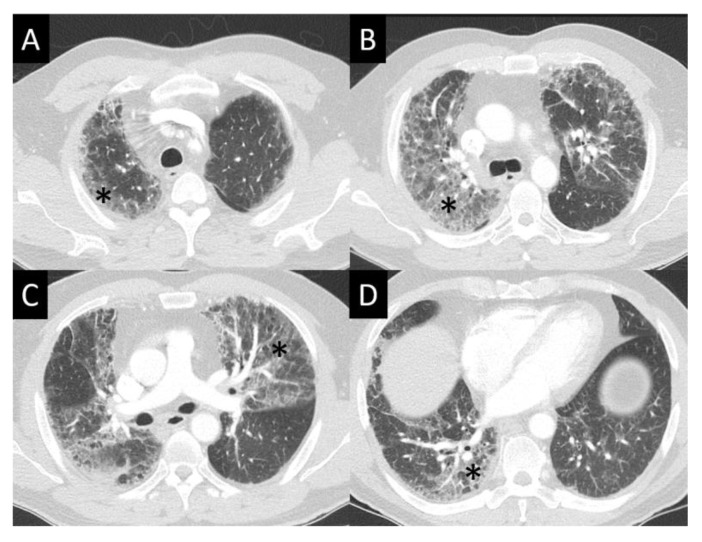
Diffuse alveolar damage (DAD). DAD is characterized by broad areas of ground glass opacity (GGO, asterisks) on CT, here seen with a geographic distribution, contrasting regions of abnormal, and normal lung (**A**–**D**).

**Figure 4:**
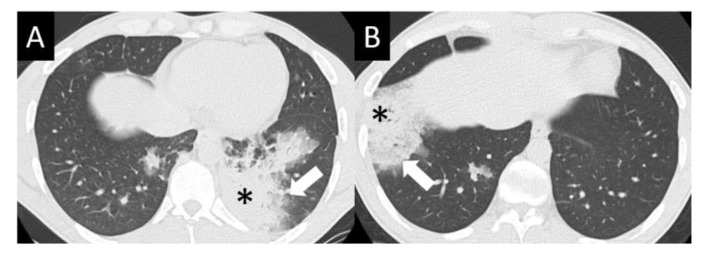
Organizing pneumonia (OP). OP is characterized by multifocal areas of peripheral consolidation (asterisks) and less dense areas of ground glass opacity (arrows). Reversed halo/atoll signs may be seen. OP often fluctuates. (**A**) Typical OP at baseline: Large volume of consolidation (asterisk), predominantly in the left lower lobe, and mild surrounding GGO (arrow). (**B**) Follow-up image 3 months later shows complete resolution of left lower lobe findings and new right lower lobe consolidation (asterisk) and GGO (arrow).

**Figure 5:**
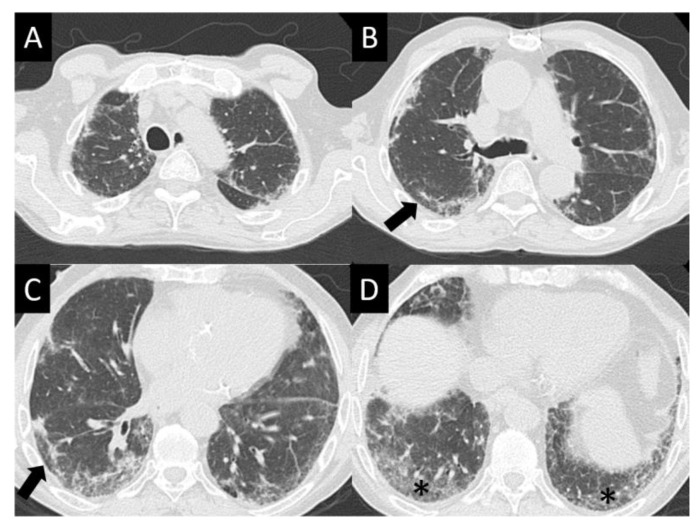
Nonspecific interstitial pneumonia (NISP). NSIP is characterized by ground glass opacity (GGO), which tends to be basal predominant with peripheral reticular opacities, seen in all images here (**A**–**D**) but best demonstrated in (**D**) (asterisk). Note sparing of the immediate subpleural lung in (**B**,**C**) (arrows), which is a hallmark of NSIP.

**Figure 6:**
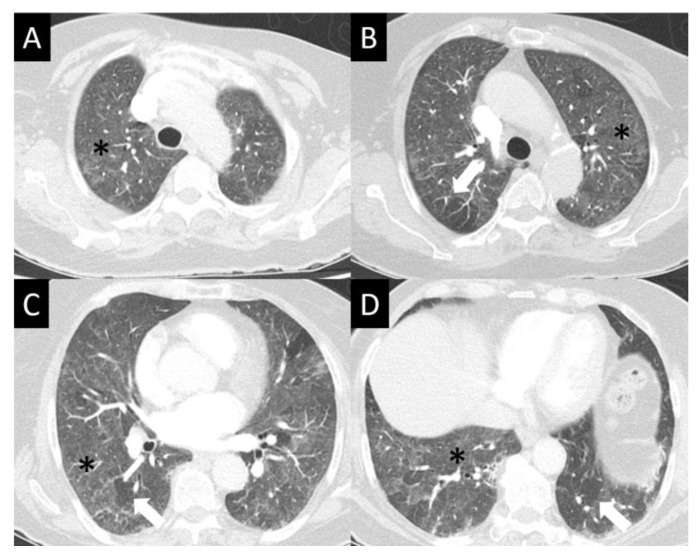
Hypersensitivity pneumonitis (HP) (**A**–**D**). HP often presents in its nonfibrotic form—seen here as subtle but diffuse ground glass opacity (GGO, asterisks), sometimes with an aspect of centrilobular nodularity. While GGO is nonspecific, mosaic lung attenuation with patchy spared lobules (arrows) is typical of the air trapping seen in HP.

DAD is the most aggressive presentation and is usually present in grade 4 DI-ILD. Features of DAD on CT include ground glass opacities (GGOs) with associated areas of consolidative opacities (Figure 3). OP is characterized by multifocal areas of GGO and peripheral consolidation. Reversed halo/atoll signs may also be seen (Figure 4). NSIP is characterized by GGO, which tends to be basal with peripheral reticular opacities (Figure 5). Features of HP on CT include diffuse GGO, ill-defined centrilobular nodules, mosaic attenuation on inspiratory images, and air trapping on expiratory CT images (Figure 6).

A diagnosis of DI-ILD requires interpretation of radiographic findings within the context of the clinical history, physical examination, and laboratory results. This may require multidisciplinary case conference discussions [70], but rapid diagnosis and upfront urgent management are critical to optimize outcomes.

### 6.2. Additional Investigations

Published guidelines recommend a number of additional procedures in the evaluation of possible DI-ILD, including sputum/blood culture, complete blood count; arterial blood gases; and respirology subspecialty consultation [1,5]. These investigations may not be essential for all patients and may be reserved for more complicated/nonresponding cases. All patients should be assessed for typical infectious processes and treated accordingly.

While blood tests alone cannot be used to diagnose DI-ILD, certain laboratory tests may play a supplementary role. Tests for nonspecific inflammatory response, tissue damage, and allergic reaction include erythrocyte sedimentation rate (ESR), C-reactive protein (CRP), and lactate dehydrogenase (LDH) [30]. Tests such as Krebs von der Lungen-6 (KL-6), pulmonary surfactant protein-A (SP-A), pulmonary surfactant protein-D (SP-D), and drug lymphocyte stimulation test (DLST) have been reported to be helpful prognostic indicators in studies [71,72,73], they are not generally available for clinical use. Tests that can aid in diagnosis and rule out infection include β-D glucan or galactomannan (invasive fungal infection), cytomegalovirus antigen, expectorated sputum bacterial smear and culture, acid-fast bacteria smear and culture, and viral and fungal polymerase chain reaction testing [63]. Bronchoscopy may be indicated if infection is suspected and has not been ruled out by less invasive investigations.

Cardiac testing for serologic markers of volume overload (beta-natriuretic peptide) may be helpful in the right clinical context.

**Summary: Key Investigations for Patients Undergoing Treatment with T-DXd**

● Conduct a history and physical examination at baseline with the focus on known T-DXd-related ILD risk factors;
● Ensure patient undergoes education and regular reminders concerning the potential adverse events associated with T-DXd and the need to immediately report symptoms, e.g., cough (particularly dry cough), shortness of breath/exertional dyspnea, fever, and unexplained fatigue;
● Pulse oximetry (SpO_2_) should be performed at each clinic assessment, and an SpO_2_ < 95% or drop of more than 4% from baseline should prompt a detailed respiratory assessment;
● Conduct baseline staging chest CT and restaging CT chest surveillance every 9–12 weeks:
  ⚬ Notify the radiologist to read the CT for both assessment of tumor response, as well as screening for ILD;
  ⚬ Conduct an HRCT promptly to confirm the diagnosis if ILD is suspected on the restaging CT but not confidently diagnosed or if ILD is clinically suspected but restaging is not required.
● Patients with infectious/inflammatory opacities on CT scan should be considered for further evaluation to elucidate the cause and severity of these abnormalities:
  ⚬ Sputum for routine culture, acid-fast bacilli, and fungus; blood work to look for markers of inflammation and infection; beta-natriuretic peptide, echocardiogram, and PFT; consultation with infectious diseases or respirology; and bronchoscopy should all be considered in the appropriate clinical scenario.

## 7. Management of T-DXd—Related ILD

Because of the possibility of rapid ILD/pneumonitis progression and because holding treatment is critical in cases of T-DXd-related events, it is important not to delay implementation of the following management steps even when diagnosis may be uncertain (Figure 2).

### 7.1. Grade 1/Asymptomatic ILD

ILD can be fatal if it is left untreated, and progression to grade 2 or higher ILD precludes continued and future treatment with T-DXd [7]. Grade 1 disease will generally be diagnosed and managed by the oncologist or oncology delegate without the need for higher-level investigation or consultation. However, if the patient has a history of previous lung disease or if there is diagnostic uncertainty, assessment by respirology may be warranted.

Grade 1 ILD requires that T-DXd be withheld until recovery to grade 0 (normalization of CT abnormalities), at which point treatment may be resumed, with the dose depending on time to resolution [7]. If resolution occurs in 28 days or fewer from onset, the original dose of T-DXd can be maintained (5.4 mg/kg–6.4 mg/kg for gastric cancer), but dose escalation is not recommended, and some clinicians may choose to dose-reduce out of an abundance of caution. If resolution takes more than 28 days, the dose is reduced to 4.4 mg/kg after a first occurrence (5.4 mg/kg for gastric cancer). If there is a second occurrence, the dose is reduced to 3.2 mg/kg (4.4 mg/kg for gastric cancer). If there is a third occurrence of pneumonitis, or if the grade 1 ILD/pneumonitis event has not resolved within 18 weeks (126 days) from the last infusion, T-DXd must be permanently discontinued.

The Canadian Product Monograph for T-DXd recommends considering corticosteroid treatment for grade 1 DI-ILD (e.g., >0.5 mg/kg/day prednisolone or equivalent until improvement, with a gradual taper over 4 weeks or longer) [7]. Until there are further data to clarify the role of steroids for grade 1 events, the steering committee recommends following this guidance, especially if any DI-ILD-related risk factors are identified. Repeat HRCT should be considered prior to each of the next two doses of T-DXd to ensure no recurrence, after which conventional chest CT scans can resume at an interval of every 9 to 12 weeks.

### 7.2. Grade 2 ILD

Symptomatic ILD that does not interfere with activities of daily living (grade 2) requires permanent discontinuation of T-DXd and prompt initiation of systemic corticosteroid treatment (e.g., ≥1 mg/kg/day prednisolone or equivalent for at least 14 days followed by a gradual taper over at least a 4-week period) [7]. PJP prophylaxis with trimethoprim/sulfamethoxazole (TMP-SMX) should be considered for all cases and is recommended for patients who are expected to be on corticosteroids at a dose of ≥20 mg for ≥1 month [74].

The patient’s symptoms should be monitored closely, with re-imaging conducted as clinically indicated. The steering committee recommends clinical reassessment 7 days after initiation of steroids and early, repeat imaging with low-dose CT scan 7 to 14 days after initiation of steroids for those with nonimproving or worsening symptoms. Precise timing of repeat chest imaging for those who are clearly responding to steroids can be determined by the treating oncologist but should occur within 4 to 6 weeks. If there is clinical or radiographic worsening (especially within 5 days of initiation of therapy), consideration should be given to increasing the dose of steroids (e.g., 2 mg/kg/day of prednisolone or equivalent) or switching to IV administration (e.g., methylprednisolone). A multidisciplinary approach to the management of these patients is indicated. At this point, additional work-up for alternative etiologies and referral to respirology should be considered.

### 7.3. Grade 3 or 4 ILD

Patients with grade 3 or higher ILD need to be hospitalized because of supplemental oxygen requirements and ventilator support. Empiric high-dose methylprednisolone IV should be promptly initiated (e.g., 500 to 1000 mg/day for 3 days), followed by at least 1.0 mg/kg/day of prednisolone (or equivalent) for at least 14 days, followed by a gradual taper over at least 4 weeks. PJP prophylaxis with TMP-SMX should be considered for all cases and is recommended for patients who are expected to be on corticosteroids at a dose of ≥20 mg for ≥1 month [74].

A multidisciplinary approach to the management of these patients is indicated. In-patient respirology consultation along with involvement of other relevant specialists should be considered, including but not limited to radiologists, intensivists, internists, and infectious disease specialists. The patient’s symptoms should be monitored closely, with re-imaging conducted as clinically indicated. The steering committee recommends daily clinical reassessment after initiation of steroids and early, repeat imaging with low-dose CT scan 7 to 14 days after initiation of steroids for those with nonimproving or worsening symptoms. Precise timing of repeat chest imaging for those who are clearly responding to steroids can be determined by the treating oncologist but should occur within 4 to 6 weeks. If there is clinical or radiographic worsening (especially within 5 days of initiation of therapy), additional work-up should be considered to explore alternative etiologies. Consider other immunosuppressants and/or treat per local practice.

**Summary: Management of T-DXd-Related DI-ILD**
*Grade 1 DI-ILD*

● Interrupt T-DXd until resolved to grade 0 (resolution of CT abnormalities), then:
  ⚬ If resolved in ≤28 days from date of onset, maintain dose (starting dose is 5.4 mg/kg (6.4 mg/kg for gastric cancer));
  ⚬ If resolved in >28 days from date of onset, reduce dose one level:
● Dose reduction with first occurrence: 4.4 mg/kg (5.4 mg/kg for gastric cancer);
● Dose reduction with second occurrence: 3.2 mg/kg (4.4 mg/kg for gastric cancer).
  ⚬ Permanently discontinue T-DXd if there is a third recurrence;
  ⚬ Permanently discontinue T-DXd if the grade 1 ILD/pneumonitis event has not resolved within 18 weeks (126 days) from the last infusion.
● Consider prednisolone ≥ 0.5 mg/kg/day or equivalent with a gradual taper over ≥4 weeks, until improvement; *
● Monitor and closely follow up in 2–7 days for onset of clinical symptoms and SpO_2_;
● Consider follow-up imaging in 1–2 weeks or as clinically indicated.

*Grade 2 DI-ILD*

● Permanently discontinue T-DXd;
● Promptly initiate corticosteroid treatment as soon as ILD/pneumonitis is suspected: *
  ⚬ A total of 1 mg/kg/day of prednisolone or equivalent for ≥14 days;
  ⚬ Gradually taper over ≥4 weeks.
● Monitor symptoms closely;
● Re-image with HRCT within 7–14 days to confirm improvement and then re-image as clinically indicated;
● If clinical or radiographic worsening or still no improvement (especially within 5 days):
  ⚬ Consider increasing dose of steroids (e.g., 2 mg/kg/day of prednisolone or equivalent), switching administration to i.v. (e.g., methylprednisolone); *
  ⚬ Reconsider additional work-up for alternative etiologies, as described above;
  ⚬ Escalate care as clinically indicated.

*Grade 3+ DI-ILD*

● Permanently discontinue T-DXd;
● Hospitalization required;
● Promptly initiate empirical high-dose methylprednisolone IV treatment: *
  ⚬ Give 500–1000 mg/day for 3 days followed by ≥1 mg/kg/day of prednisolone (or equivalent) for ≥14 days;
  ⚬ Gradually taper over ≥4 weeks
● Re-image with HRCT within 7–14 days to confirm improvement and then re-image as clinically indicated;
● If clinical or radiographic worsening or still no improvement (especially within 5 days):
  ⚬ Reconsider additional work-up for alternative etiologies, as described above;
  ⚬ Consider other immunosuppressants and/or treat per local practice.
● Consider involvement of respirology or internal medicine.

* Consider PJP prophylaxis with TMP-SMX for all patients—recommended for patients who are expected to be on corticosteroids at a dose of ≥20 mg for ≥1 month.

## 8. Conclusions

With the increase in novel therapies for cancer, the occurrence of DI-ILD is increasing and is a significant clinical challenge, as it requires early, presymptomatic diagnosis to avoid severe toxicity. Given the positive clinical trial results to date, T-DXd has the potential for widespread use in Canada in breast cancer and a variety of other malignancies. It is reasonable to expect that as the routine use of T-DXd increases, higher rates and grades of DI-ILD will be observed in real-world clinical care compared to that observed in randomized, controlled clinical trials. A proactive monitoring strategy aimed at early detection, along with a clear and rapidly initiated treatment algorithm, is critical to optimize clinical outcomes and minimize the risk of high-grade or fatal ILD [5,30].

T-DXd is unique among DI-ILD, because the occurrence of grade 2 or higher DI-ILD necessitates permanent cessation of the drug and even grade 1, asymptomatic findings require treatment interruption. To optimize treatment outcomes and minimize the risk of life-threatening T-DXd-related ILD, diligence in proactive monitoring is needed to ensure that potential cases of DI-ILD are identified and resolved in the earliest, asymptomatic stage (grade 1). This may require a multidisciplinary approach, involving the oncologist, radiologist, respirologist, and allied health care providers. Ongoing patient education ensuring awareness of the risks and the importance of reporting any potential symptoms of DI-ILD as soon as they occur is critical.

The need for closer monitoring of patients undergoing treatment with T-DXd, including a greater frequency of CT scans and the use of HRCT to confirm suspected cases of ILD, will translate to increased use of health care resources. A focus on screening, monitoring, and managing grade 1 ILD effectively will likely reduce the risk of progression to higher-grade lung toxicity. A novel and dedicated model of multidisciplinary care in oncology (medical oncologist, radiologist, and respirologist) in managing DI-ILD in breast cancer and other disease sites will likely be warranted as T-DXd, as well as other potentially pneumotoxic agents, become widely available in Canada (Table 4).

## Figures and Tables

**Table 1:** Key efficacy results in the T-DXd DESTINY trials.

Study	*n*	Population	Design	Median PFS, Months	Median OS, Months	Response Rate (RR), %	Duration of Response, Months
DESTINY-Breast01 [1,8]	184	HER2-positive metastatic breast cancer with previous treatment with trastuzumab emtansine	Open-label Phase 2 Single arm	16.4	29.1	Overall RR 60.9	14.8
DESTINY-Breast02 [2]	608	HER2-positive metastatic breast cancer with previous treatment with trastuzumab emtansine	Open label Phase 3 Randomized (T-DXd vs. investigator’s choice of treatment)	17.8 T-DXd vs. 6.9 investigator’s choice	39.2 T-DXd vs. 26.5 investigator’s choice	Objective RR 69.7 T-DXd vs. 29.2 investigator’s choice	19.6 T-DXd vs. 8.3 investigator’s choice
DESTINY-Breast03 [3]	524	HER2-positive metastatic breast cancer previously treated with trastuzumab and a taxane	Open label Phase 3 Randomized (T-DXd vs. T-DM1)	28.8 T-DXd vs. 6.8 T-DM1	Not reached	Overall RR 79 T-DXd vs. 35 T-DM1	36.6 T-DXd vs. 23.8 T-DM1
DESTINY-Breast04 [4]	557	HER2-low * metastatic breast cancer patients who received one or two previous lines of chemotherapy	Open label Phase 3 Randomized (T-DXd vs. physician’s choice of chemotherapy)	9.9 T-DXd vs. 5.1 physician’s choice of chemotherapy	23.4 T-DXd vs. 16.8 physician’s choice of chemotherapy	Objective RR 52.3 T-DXd vs. 16.3 physician’s choice of chemotherapy	10.7 T-DXd vs. 6.8 physician’s choice of chemotherapy
DESTINY-Gastric01 [9]	187	HER2-positive advanced gastric cancer	Open label Phase 2 Randomized (T-DXd vs. physician’s choice of chemotherapy)	5.6 T-DXd vs. 3.6 physician’s choice of chemotherapy	12.5 T-DXd vs. 8.4 physician’s choice of chemotherapy	Objective RR 51 T-DXd vs. 14 physician’s choice of chemotherapy	11.3 T-DXd vs. 3.9 physician’s choice of chemotherapy
DESTINY-Gastric02 [10]	79	HER2-positive unresectable or metastatic gastric/GEJ cancer	Phase 2 Single arm	5.6	12.1	Objective RR 41.8	8.1
DESTINY-Lung01 [11]	91	HER2-mutant unresectable or metastatic NSCLC	Phase 2 Single arm	8.2	17.8	Objective RR 55	9.3

* Low expression of HER2 was defined as a score of 1+ on immunohistochemical (IHC) analysis or as an IHC score of 2+ and negative results on in situ hybridization. GEJ: gastroesophageal junction; NR: not reported; NSCLC: nonsmall cell lung cancer; T-DXd: trastuzumab deruxtecan; T-DM1: trastuzumab emtansine.

**Table 2:** Incidence and severity of interstitial lung disease/pneumonitis associated with various molecular targeting and immune checkpoint inhibitor anticancer treatments other than trastuzumab deruxtecan (T-DXd).

Treatment	Tumor Types	Number of Studies (Number of Patients)	Any Grade ILD, *n* (%)	Grade 5 ILD, *n* (%)
**Anti-HER2**				
Trastuzumab [42]	HER2-positive advanced or unresectable/metastatic breast cancer	8 (1642)	162 (9.9)	3 (0.2)
Lapatinib [42]	HER2-positive advanced or metastatic breast cancer	4 (4470)	8 (0.2)	0
T-DM1 [42]	HER2-positive advanced or metastatic breast cancer	3 (3290)	15 (0.5)	6 (0.2)
SYD985 [42,43] ^a^	HER2-expressing ^b^ locally advanced or metastatic breast, gastric, urothelial, or endometrial cancer	1 (185)	4 (2.2)	1 (0.5)
SYD985 [44]	HER2-positive locally advanced or metastatic breast cancer	1 (291)	NR (7.6)	2 (0.7)
ARX788 [45]	HER2-positive advanced gastric and gastroesophageal junction cancer	1 (23)	NR	0
ARX788 [46] ^c^	HER2-positive advanced breast cancer	1 (69)	NR (4.3) ^c^	NR
ARX788 [46] ^c^	HER2-positive advanced solid tumors	1 (34)	NR (2.9) ^c^	NR
**TKI and/or EGFR inhibitor**				
Gefitinib [34] ^d^	*EGFR*-mutated NSCLC	2 (201)	10 (5.0)	2 (1.0)
Gefitinib [35] ^d^	NSCLC	1 (330)	8 (2.4)	6 (1.8)
Erlotinib [36] ^d^	Recurrent/advanced NSCLC	NA (3488)	158 (4.5)	55 (1.6)
Crizotinib, ceritinib, alectinib, brigatinib, lorlatinib, TSR-011, ASP3026, or ensartinib [37]	NSCLC	NA (4943)	37 (0.7)	5 (0.1) ^e^
Cetuximab [38] ^d^	Head and neck squamous cancer	NA (201)	9 (4.5)	1 (0.5)
Osimertinib [39] ^d^	*EGFR*-mutated inoperable or recurrent NSCLC	NA (3578)	231 (6.5) ^f^	29 (0.8)
Alectinib, ceritinib, crizotinib, or brigatinib [40] ^g^	Advanced NSCLC	18 (2261)	NR (2.14)	NR (0.22)
Erlotinib, gefitinib, afatinib, or osimertinib [41] ^g^	Advanced NSCLC	144 (15,713)	NR (1.12)	NR (0.20)
**Immune checkpoint inhibitor**				
Nivolumab, pembrolizumab, atezolizumab, or durvalumab [52] ^d^	Lung cancer	NA (102)	19 (18.6)	4 (3.9)
Nivolumab or pembrolizumab (PD-1 inhibitors) [53] ^a,g^	NSCLC	12 (3232)	NR (3.6)	7 (NR)
Atezolizumab, durvalumab, or avelumab (PD-L1 inhibitors) [53] ^a,g^	NSCLC	7 (1806)	NR (1.3)	0
Anti-PD-1 monotherapy [54] ^d^	NSCLC	NA (138)	20 (14.5)	3 (2.2)
Nivolumab [55] ^d^	Recurrent or advanced NSCLC	2 (111)	8 (7.2)	1 (0.9)
PD-L1 inhibitors [56] ^a,g^	Melanoma, NSCLC, or renal cell carcinoma	20 (4496)	NR (2.7)	NR
CTLA-4, PD-1, or PD-L1 inhibitors [57]	NSCLC, melanoma, cavum, Hodgkin’s lymphoma, or UCNT	NR (1862)	64 (3.5)	6 (0.3)
**CDK4/6 Inhibitors**				
Abemaciclib [48]	Metastatic breast cancer	3 (900)	NR (3.2)	NR (0.4)
Palbociclib [49]	HR-positive, HER2-negative advanced breast cancer	3 (872)	6 (0.69)	0 (0)
Ribociclib [50]	HR-positive, HER2-negative advanced breast cancer	3 (1153)	NR (1.6)	NR (0.1)

CTLA-4: cytotoxic T-lymphocyte antigen-4; EGFR: epidermal growth factor receptor; HER2: human epidermal growth factor receptor 2; HR: hormone receptor; ILD: interstitial lung disease; NA: nonapplicable; NR: not reported; NSCLC: nonsmall cell lung cancer; PD-1: programmed cell death protein 1; PD-L1: Programmed cell death ligand 1; SYD985: trastuzumab duocarmazine; T790M: methionine substitution for threonine at amino acid position 790; T-DM1: trastuzumab emtansine; TKI: tyrosine kinase inhibitor; UCNT: undifferentiated carcinoma of nasopharyngeal type. ^a^ The study was specific to pneumonitis. ^b^ HER2 expression was required for eligibility for the dose-expansion phase of this study (*n* = 146). Patients were eligible for the dose-escalation phase (*n* = 39) regardless of their HER2 status. ^c^ Percentages were only reported for grade > 3 pneumonitis. ^d^ Study conducted in Japanese patients only. ^e^ ILD grade information was unavailable for 11 patients who had ILD events. ^f^ The ILD cases reported here were those that were adjudicated by an ILD expert committee; there were 245 patients with ILD reported by their attending physicians. ^g^ The pooled incidence of pneumonitis was reported. Reprinted from *Cancer Treatment Reviews*, Vol. 106, Swain S.M., et al. [5], Multidisciplinary clinical guidance on trastuzumab deruxtecan (T-DXd)-related interstitial lung disease/pneumonitis-Focus on proactive monitoring, diagnosis, and management, 102378., Copyright (2022), with permission from Elsevier.

**Table 3:** Rates of interstitial lung disease (ILD)/pneumonitis in the T-DXd DESTINY clinical trials.

Study	*n* *	Population	ILD Incidence (T-DXd Arm), %		
			Any Grade	Grade 1 or 2	Grade 5
DESTINY-Breast01 [1]	184	HER2-positive metastatic breast cancer with previous treatment with trastuzumab emtansine	13.6	10.9	2.2
DESTINY-Breast02 [2]	404	HER2-positive metastatic breast cancer with previous treatment with trastuzumab emtansine	10.4	9.2	0.5
DESTINY-Breast03 [3]	257	HER2-positive metastatic breast cancer previously treated with trastuzumab and a taxane	15.2	4.3	0
DESTINY-Breast04 [4]	371	HER2-low ^†^ metastatic breast cancer patients who received one or two previous lines of chemotherapy	12.1	10	0.8
DESTINY-Gastric01 [9]	125	HER2-positive advanced gastric cancer	9.6	7.2	0
DESTINY-Gastric02 [10]	79	HER2-positive unresectable or metastatic gastric/GEJ cancer	10.1	7.6	2.5
DESTINY-Lung01 [11]	91	HER2-mutant unresectable or metastatic NSCLC	26.4	19.8	2.2

* T-D0058d arm. ^†^ Low expression of HER2 was defined as a score of 1+ upon immunohistochemical (IHC) analysis or as an IHC score of 2+ and negative results on in situ hybridization. GEJ: gastroesophageal junction; NR: not reported; NSCLC: nonsmall cell lung cancer; T-DXd: trastuzumab deruxtecan; T-DM1: trastuzumab emtansine.

**Table 4:** Roles of the multidisciplinary team in the management of DI-ILD.

Medical Oncologist	Radiologist	Respirologist
Taking a detailed history for relevant risk factors Monitoring for potential signs and symptoms Notifying the radiologist that the patient is taking a drug that could lead to DI-ILD Treatment of all stages of ILD/pneumonitis Referral to respirology if required	Knowing that the patient is taking a drug with known pulmonary toxicities Prompt communication of any findings suspicious of ILD/pneumonitis	Evaluating patients with pre-existing lung comorbidities (asthma, COPD, ILD, etc.) before starting treatment with T-DXd Conducting baseline PFTs for patients with a history of lung comorbidities (if not already performed) Assisting in treatment decisions for grade 2 or higher ILD/pneumonitis
